# Association between Adherence to the Mediterranean Diet and Anthropometric and Health Variables in College-Aged Males

**DOI:** 10.3390/nu14173471

**Published:** 2022-08-24

**Authors:** Pablo Prieto-González, Jorge Sánchez-Infante, Luis Miguel Fernández-Galván

**Affiliations:** 1 Health and Physical Education Department, Prince Sultan University, Riyadh 11586, Saudi Arabia; 2 Performance and Sport Rehabilitation Laboratory, Faculty of Sport Sciences, University of Castilla-La Mancha, 45071 Toledo, Spain; 3 Department of Physical Education, Sport, and Human Movement, Autonomous University of Madrid, 28049 Madrid, Spain

**Keywords:** Mediterranean diet, anthropometric parameters, health variables, KIDMED index

## Abstract

The present study aimed to verify the association between adherence to the Mediterranean diet (MD) and anthropometric and health variables. Four-hundred-and-ninety-five college-aged males aged 18–25 participated in this cross-sectional research. The KIMED (Mediterranean Diet Quality Index for children and adolescents) was used to assess the adherence to MD. The following variables were also assessed: body mass (BM), height (HE), body mass index (BMI), body fat percentage (%FAT), lean mass (LEAN), abdominal girth (AG), waist-to-hip ratio (WHR), oxygen saturation (SPO2), systolic blood pressure (SBP), diastolic blood pressure (DBP), double product (DP), and fasting blood glucose (GLU). The results showed that adherence to MD presented a strong negative correlation with most of the anthropometric parameters (BM: *r* = −0.571; BMI: *r* = −0.614; %FAT: *r* = −0.558; and AG: *r* = −0.564), a moderate or weak correlation with most of the health variables (GLU: *r* = −0.407; SBP: *r* = −0.238; DBP: *r* = −0.217, and DP: *r* = −0.265) and LEAN (*r* = −0.497), and a very weak correlation with WHR (*r* = −0.090). Many anthropometric parameters (BM, BMI, %FAT, LEAN, AG, WHR) present significant correlations with health variables (SBP, DBP, DP, and GLU). We conclude that greater adherence to Mediterranean diet is associated with healthier values of the selected anthropometric and health parameters. Since most of the anthropometric and health parameters present significant correlations among themselves, this finding could be useful in medical diagnosis, health monitoring, and risk detection. Based on the level of adherence to Mediterranean diet and the KIDMED found in the present study, and considering the prevalence of obesity in the Middle East, it is imperative to implement nutritional interventions with the target population of this research to prevent nutrition-related diseases and promote public health.

## 1. Introduction

Nowadays, the Mediterranean diet (MD) is one of the healthiest dietary patterns. It provides an adequate quantity and quality of nutrients to improve health conditions and prevent diseases [1,2]. In addition, it has been the most studied and recognized dietary approach in the world during the last decades, since the 1960s [2,3]. MD was declared an Intangible Cultural Heritage of Humanity in 2010 by UNESCO for being not only a food pattern but also a social, cultural, and philosophical model of relating with people and sharing the table with family and friends. It involves the preservation of traditional craftsmanship in agriculture, livestock and fishing, and maintaining an active lifestyle. It is a model of biodiversity and sustainability due to its low environmental footprint. MD originated in Mediterranean countries over centuries, particularly Spain, Italy, and Greece [3,4,5,6]. It is the result of the interaction between dietary patterns and the geographic, climatological, orographic, cultural, and environmental conditions of those countries, wherein the existence of olive trees is a common factor [7,8].

MD is based on a regular and generous consumption of seasonal plant-based foods such as fruits, vegetables, nuts, and whole grains. Olive oil is the main source of fat. The intake of fish, dairy products, poultry, and eggs is moderate, whereas red meat and sausages are consumed infrequently and in small quantities [9]. Thus, MD is rich in monounsaturated and polyunsaturated fats, omega-3 fatty acids, fiber, natural antioxidants, and phytoactive compounds. It provides the necessary minerals, vitamins, proteins, and energy, and is low in saturated and trans fats [10,11,12]. As a result of the intake of these micro and macronutrients, MD has a preventive effect on the acquisition of non-communicable diseases such as cancer, type 2 diabetes, metabolic syndrome, cardiovascular diseases, obesity, fatty liver, hypertension, fragility syndrome in older adults, Alzheimer’s disease, dementia, and depression. It also improves the symptoms of heart disease, is useful for weight loss, and increases life expectancy [11,13,14,15,16,17,18,19,20,21,22].

Unfortunately, adherence to MD has declined worldwide, including in Mediterranean countries [23], and among young adults [24]. This resulted in the adoption of less healthy eating habits that led to a deterioration in nutritional quality [25,26]. In this context, MD has been a research subject in longitudinal and cross-sectional studies to promote healthier nutrition. Most have been carried out in Mediterranean European countries, and a smaller number in North African and Middle Eastern countries [27]. In the case of college-aged subjects—although few studies have been devoted to analyze their eating habits and diet quality—the existing evidence indicates that they follow an unhealthy dietary pattern [28]. However, the results of these existing studies are also contradictory in certain cases. As for the adherence to MD, the percentage of subjects in this age group with optimal adherence ranges from 9.5% to 58% [24,29,30,31,32,33,34,35]. Likewise, while some studies have found a clear association between adherence to MD and anthropometric parameters [36,37], this association between variables has not been observed in other research [38,39].

Furthermore, many existing articles examined the adherence to MD [24,34], and some of them also show how this adherence correlates to selected anthropometric parameters such as BMI (body mass index), WHR (waist-to-hip ratio) [30,40], or self-perceived health [31]. However, there is a lack of research in young adults analyzing the association between adherence to MD with a comprehensive number of biomarkers and anthropometric variables (i.e., body fat percentage, lean mass, oxygen pulse, blood pressure, fasting blood glucose) and using large randomized samples. Only a few studies examined this correlation, but they were conducted mainly with middle-aged adults and senior citizens instead of young adults [41,42]. 

In non-Mediterranean countries, MD is being promoted because its benefits are increasingly recognized. This has been accompanied by increased scientific interest in MD [27]. However, in the case of the Middle East—despite its geographical and cultural proximity to Mediterranean countries—very few studies have analyzed the association between adherence to MD and anthropometric and health variables [43,44]. Nevertheless, what the existing scientific evidence does reveal in Middle Eastern countries is bad dietary habits, high levels of obesity, and the need for nutritional interventions to improve community health, well-being, and disease prevention [45,46,47,48]. Against this background, further research must be conducted in the Middle East with college-aged students to verify whether adherence to MD is associated with health and anthropometric parameters. In countries with high levels of obesity, it is essential to know what percentage of this age group has adequate eating habits, and if new healthy behaviors must be acquired [28,49]. Such studies should ideally be carried out by examining a comprehensive number of biomarkers and with large randomized samples. In this regard, to assess adherence to MD, the most widely used instrument is the KIDMED (Mediterranean Diet Quality Index for children and adolescents) questionnaire. The validity of this instrument has been confirmed in previous studies in the Mediterranean and non-Mediterranean countries for children, adolescents, and young adults [50,51]. Therefore, the present study aimed to verify the association between adherence to MD and anthropometric and health variables in Saudi college-aged males.

## 2. Materials and Methods

A cross-sectional descriptive observational study was carried out. The STROBE (strengthening the reporting of observational studies in epidemiology) guidelines were followed.

### 2.1. Subjects

A total of 495 college-aged individuals were included in the current research. They were duly informed of the objectives, benefits, and risks of participating in the present study and signed an informed consent form indicating their willingness to be included in it. They were also instructed that they could withdraw from the study without penalty. Subjects were granted anonymity when handing the data collected and informed that it would be used only for scientific purposes. Moreover, they receive no academic or monetary compensation for their contribution. The inclusion criteria were:

(a) Male
(b) Aged 18–25 years
(c) Resident in Riyadh
(d) Do not suffer from nutritional (i.e., anorexia, bulimia), traumatic (i.e., fractures, fissures), genetic (i.e., hemophilia, cystic fibrosis), endocrine (i.e., diabetes, metabolic syndrome), mental (i.e., schizophrenia, bipolar disorder), or degenerative (i.e., sclerosis, osteoarthritis) diseases.

The study was held in Riyadh between 15 May and 30 June 2022. A stratified random sample from the 15 districts that make up the city was used for subject selection. Potential participants were contacted and invited to participate in the current research through Riyadh municipality forum groups available on social media (WhatsApp and Facebook Messenger). Once 600 volunteers expressed their willingness to participate in the study, all of them were summoned to attend the tests and fill out the questionnaire. Finally, a total of 495 subjects completed all assessments. To estimate the sample size required, the following equation [52]:

n = Z2p × qN/e2 (N − 1) + Z2p q (2)(1)

where n = sample size; N = population size; Z = confidence level; p = probability of success; q = probability of failure; e = confidence interval. The confidence level was set at 95%, the confidence interval at 5%, and the probability of success at 50%. Subsequently, it was determined that the minimum number of subjects needed to have a representative sample of the population studied was 385.

### 2.2. Assessments

#### 2.2.1. Adherence to the Mediterranean Diet

The KIMED index was used to assess the adherence to MD. This questionnaire is based on the principles of the MD pattern. The validity and reliability of this instrument to measure the adherence to MD of college-aged individuals in non-Mediterranean countries has been previously confirmed [50]. The KIDMED consists of 16 items used to identify whether the dietary habits and amounts ingested are adequate. Some questions reflect adherence to MD and assign one point, whereas other questions denote non-adherence to MD and subtract one point. In this way, each respondent obtains a score that is classified into three levels: (a) (1) ≥8 points: optimal diet quality; (b) 4–7 points: improvements are needed to adjust intake to Mediterranean patterns; (c) ≤3 points: very low diet quality [51,53,54]. The data were collected using the digital support of Google Forms. This process was conducted in the presence of the researchers, who explained in detail to all respondents the meaning of the 16 questions and clarified doubts. 

The KIDMED questionnaire was applied again to all subjects to verify the reliability of the measurement. That is, a test–retest method was used. The interval between both measurements was three weeks, which is the optimal recommended time [55]. The test–retest coefficient was calculated using SPSS and choosing the two-way random model. The interclass correlation coefficient obtained was 0.97, which reflects excellent reliability [56]. Subsequently, once the questionnaires were submitted, the researchers met to verify that the information collected was not flagged or incomplete, and when applicable, amend possible errors. In any case, the incomplete submission of the questionnaire was largely avoided by the functionality of Google Forms since it does not allow the partial submission of responses.

#### 2.2.2. Anthropometric and Health Variables

Assessments were made following the indications of the ISAK [57], and using a room with a temperature between 20 and 22 degrees. The measurements were conducted between 8 am and 10 am on an empty stomach and were taken in the right hemisphere. Each parameter was assessed three times (except the blood fasting glucose), and the value finally recorded was the median of those three measurements. The researchers previously calibrated all the material with a sample of 30 subjects. Body mass (BM), height (HE), and body mass index (BMI) were measured with a Seca digital column scale (Hamburg, Germany). BM was recorded to the nearest 0.1 kg, and HE to the nearest 0.1 cm. The three previous measurements were taken with the subjects barefoot and in light clothing. Body fat percentage (%FAT) was estimated using the following equation [58]:

%FAT = [(Σ of abdominal, supraspinal, subscapular, triceps, quadriceps, and calf skinfolds) × 0.143] + 4.56(2)

To measure the skin folds, a FG1056 Harpenden skinfold (Sussex, United Kingdom) caliper was used. Lean mass (LEAN) was calculated with the following equation:

LT = total weight (kg) − at mass (kg)(3)

Abdominal girth (AG) and WHR were evaluated using a Seca 203 Ergonomic Circumference Measuring Tape (Hamburg, Germany), and it was calculated through the following equation:

WHR = waist circumference (cm)/hip circumference (cm)(4)

Oxygen saturation (SPO2) was measured on the index finger, with the patient seated and calm, using a BPL Smart Oxy Lite Pulse Oximeter (Arakere, Bangalore, India). Systolic and diastolic blood pressure (SBP and DBP) and heart rate (HR) were assessed with the subject seated and relaxed using an Omron Upper Arm Gold Blood Pressure Monitor (Kyoto, Japan). The three measurements were taken with a time interval of 1 min [59]. The double product (DP), which is an index of cardiac oxygen consumption, was estimated using the following equation:

Double Product = Systolic Blood Pressure × heart rate(5)

Finally, fasting blood glucose (GLU) was measured with a Benecheck 3 in 1 Meter Kit (New Taipei City, 242 Taiwan). Each of the above variables was measured by the same researcher.

### 2.3. Ethical Clearance

This study was conducted in accordance with the ethical principles set out in the Helsinki Declaration. It was also approved by the Institutional Review Board of the Bioethics Committee at Prince Sultan University in Riyadh, Saudi Arabia (PSU IRB-2022-06-0115).

### 2.4. Statistical Analysis

The results are presented using the format mean SD (standard deviation). The data’s normality and homoscedasticity were verified using Kolmogorov–Smirnov and Levine’s test, respectively. The associations between variables were examined using the Pearson correlation coefficient, and the results were interpreted as follows: *r* = 0 null correlation; 0.01 ≤ *r* ≤ 0.09 very weak; 0.10 ≤ *r* ≤ 0.29 weak; 0.30 ≤ *r* ≤ 0.49 moderate; 0.50 ≤ *r* ≤ 0.69 strong; and *r* ≥ 0.70 very strong [60]. The one-way ANOVA test was conducted to compare subgroups or cohorts. When statistically significant *p* values were found, Tukey’s post hoc test was performed. The effect size was calculated using the partial eta squared (ηp2). Values of ηp2 = 0.01, ηp2 = 0.06, and ηp2 = 0.14 were considered as small, medium, and large effect sizes, respectively [61]. A stepwise multiple linear regression analysis was conducted to understand the relationship between the different variables included in the present study. For this purpose, KIDMED was used as the dependent variable and the anthropometric and health variables as independent variables. The level of significance established was set at *p* < 0.05. The statistical analysis was performed using the program IBM SPSS V.26^®^ computing (IBM Corp., Armonk, NY, USA).

## 3. Results

The characteristics of study participants and the results they obtained in the anthropometric and health assessments are shown in Table 1.

Of the 495 study participants, 6.46% (n = 32) showed a low level of adherence to the Mediterranean diet, 62.62% (n = 310) a medium adherence, and 30.91% (n = 153) a high adherence. The average KIDMED index was 6.41 (1.87) for the whole sample, and for the cohorts that present a low, medium and high level of adherence to MD was 2.80 (0.20), 5.75 (0.15), and 8.54 (0.13), respectively. Furthermore, once the normality and homoscedasticity of the data had been verified, Pearson’s correlation analysis was conducted (see Table 2), and the existence of a significantly negative correlation between adherence to the MD and the following variables was observed: BM, BMI, %FAT, LEAN, SBP, DBP, GLU, AG, WHR, and DP. In contrast, a significantly positive correlation was observed between AGE and BM, LEAN, DBP, WHR. Similarly, BM was significantly positively related to BMI, %FAT, LEAN, SBP, DBP, GLU, AG, WHR, and DP. A significant positive correlation was observed between BMI and %FAT, LEAN, SBP, DBP, GLU, AG, WHR, and DP. The variable of %FAT presented a significantly positive correlation with LEAN, SBP, DBP, GLU, AG, and DP. SBP was significantly positively correlated with DBP, GLU, AG, WHR, and DP. DBP presented a significantly positive correlation with GLU, AG, and DP. GLU was significantly positively correlated with AG, WHR, and DP. AG presented a significantly positive correlation with WHR, and DP. Finally, DP had a significant positive correlation with WHR.

**Figure 1:**
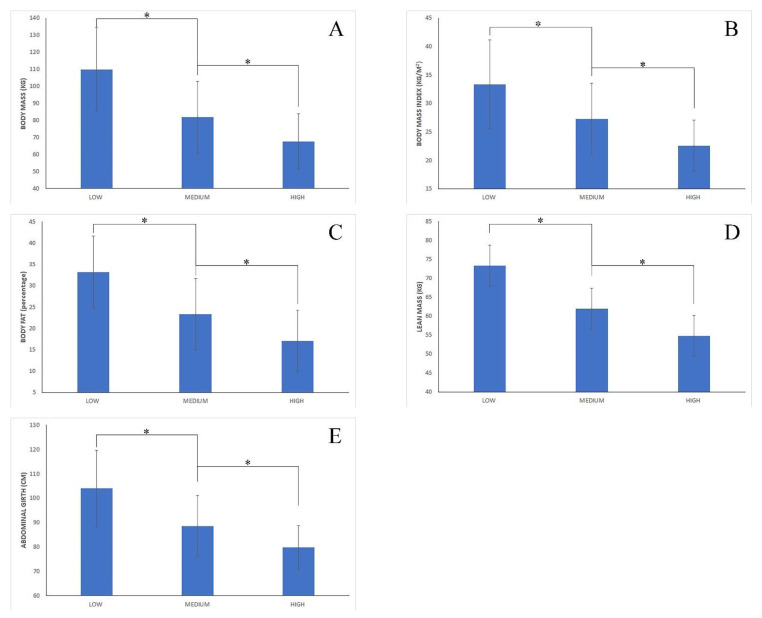
Anthropometric variables. Comparison between cohorts based on their level of adherence to the Mediterranean diet (low vs. medium vs. high). (**A**) Body mass (Kg); (**B**) body mass index (Kg/m^2^); (**C**) body fat percentage (%); (**D**) lean mass (kg); (**E**) abdominal girth (cm); *: significant difference (*p* < 0.05).

**Figure 2:**
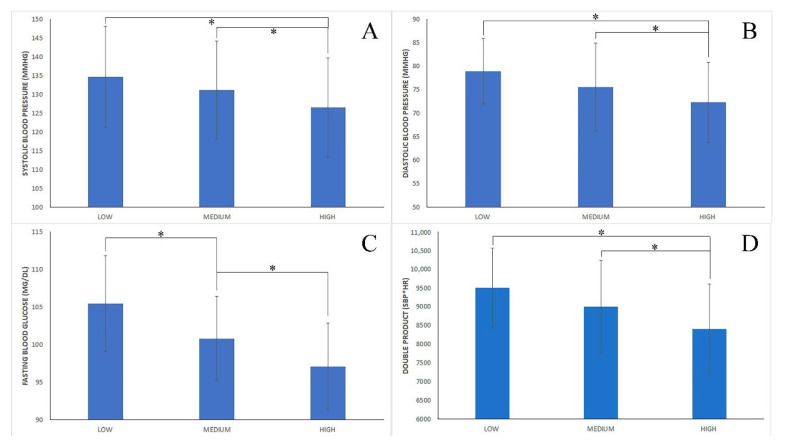
Health variables. Comparison between cohorts based on their level of adherence to the Mediterranean diet (low vs. medium vs. high). (**A**) Systolic blood pressure (mmHg); (**B**) diastolic blood pressure (mmHg); (**C**) fasting blood glucose (mg/dL); (**D**) double product (SBP × HR); *: significant difference (*p* < 0.05).

Comparisons between different levels of adherence to the Mediterranean diet are shown in Figure 1 for anthropometric variables and in Figure 2 for health variables. The one-way ANOVA test showed the existence of a main effect of the following variables: BM (*p* < 0.0001; ηp2 = 0.211); BMI (*p* < 0.0001; ηp2 = 0.249); %FAT (*p* < 0.0001; ηp2 = 0.208); LEAN (*p* < 0.0001; ηp2 = 0.147); AG (*p* < 0.0001; ηp2 = 0.210); SBP (*p* < 0.0001; ηp2 = 0.034); DBP (*p* = 0.002; ηp2 = 0.026); GLU (*p* < 0.0001; ηp2 = 0.134); and DP (*p* < 0.0001; ηp2 = 0.197). The posterior Tukey´s post hoc test revealed that subjects´ with low adherence to MD presented a significantly higher BM (*p* < 0.0001; F = 19.28–36.68); BMI (*p* < 0.0001; F = 6.47–11.60); %FAT (*p* < 0.0001; F = 6.33–13.34); LEAN (*p* < 0.0001; F = 6.39–16.33); AG (*p* < 0.0001; F = 10.16–20.52); and GLU (*p* < 0.0001; F = 2.14–7.13) than those subjects with medium adherence to MD. Similarly, subjects with low adherence to MD presented a higher BM (*p* < 0.0001; F = 32.94–51.16); BMI (*p* < 0.0001; F = 11.05–16.42); %FAT (*p* < 0.0001; F = 12.46–19.80); LEAN (*p* < 0.0001; F = 13.37–23.77); AG (*p* < 0.0001; F = 18.84–29.59); SBP (*p* = 0.004; F = 2.11–14.08); DBP (*p* = 0.004; F = 1.54–9.74); GLU (*p* < 0.0001; F = 5.79–11.01); and DP (*p* < 0.0001; F = 541.04–1654.01) than those subjects with high adherence to MD. Finally, subjects with medium adherence to MD presented a significantly higher BM (*p* < 0.0001; F = 9.43–18.69); BMI (*p* < 0.0001; F = 3.33–6.06); %FAT (*p* < 0.0001; F = 4.43–8.16); LEAN (*p* < 0.0001; F = 4.56–9.85); AG (*p* < 0.0001; F = 6.09–11.55); SBP (*p* = 0.001; F = 1.64–7.63); DBP (*p* = 0.024; F = 0.23–4.40); GLU (*p* < 0.0001; F = 2.43–5.09); and DP (*p* < 0.0001; F = 309.17–874.86) than those subjects with high adherence to MD. Moreover, no significant effect was found for the following variables: AGE, HE, SPO2, and WHR.

The multiple regression analysis conducted originated two models (see Table 3). According to model 1, BMI is a predictor variable of KIDMED, and based on model 2 BMI and %FAT are predictors of KIDMED. 

## 4. Discussion 

This research analyzed the association between adherence to MD and anthropometric and health variables. To our knowledge, this research is one of the few studies that has analyzed the mentioned association, including a large stratified sample and a comprehensive number of anthropometric and health variables. It is also the only study to do so in the Middle East region with college-age individuals. One of the main findings of the present study was that 6.46% of the study participants showed a low level of adherence to MD, 62.62% a medium adherence, and 30.91% a high adherence. If we compare these data with studies carried out with subjects of similar ages wherein the KIDMED index was used, we can appreciate the level of adherence is similar to that registered by de la Montaña et al. (2012) [30], but higher than the level of adherence reported by Durá-Travé et al. (2011) [29] and Padial-Ruz et al. (2018) [40], and lower than the level of adherence observed by Navarro-González et al. (2016) [32]. The KIDMED index of the present study was 6.41(1.87). This poor adherence to MD may be caused by factors such as industrialization, delocalization of food production, urbanization, and globalization, which condition eating habits, lifestyles and food preparation [23]. In the specific case of young adults, the low adherence found in this research and in similar studies could be associated with age-related factors, since after reaching the age of 18, individuals may leave their family environment and may abandon family habits due to changes in the organization of their studies or to starting work. Thus, at this age, individuals often opt for ready-made meals for reasons of convenience, lack of time, lack of culinary habits, and lack of knowledge of where to buy food, which is often consumed without a common table and sometimes in front of screens [24]. This way of eating, which is very common in Northern and Central Europe and North America, is known as the Western diet [25]. In contrast to MD, the Western diet is based on the intake of highly processed and energy-dense foods.

Therefore, considering KIDMED figures found in the present research, there is significant room for improvement in the eating habits of the study participants. To this end, Durá-Travé et al. (2011) [29] consider that it is advisable to develop nutrition education programs in the academic curriculum of the Universities. In this sense, some studies have verified that acquiring knowledge of nutrition and healthy lifestyles is associated with greater adherence to MD in university students [62,63]. The level of adherence found in the present study is similar to that obtained by Shatwan et al. (2021) [43] in adults aged 20 to 55 years in Gulf countries. However, it should be borne in mind that the present research was carried out in an urban area, and according to previous studies, rural populations present higher levels of adherence to MD [64].

Another important finding of the present study is the significant negative correlation found between adherence to MD and most of the variables analyzed. This correlation is strong with most anthropometric parameters (BM, BMI, %FAT, and AG), moderate or weak with many health variables (GLU, SBP, DBP, and DP) and LEAN, and very weak in the case of WHR. In addition, it should also be stressed that BMI and %FAT are predictor variables of KIDMED. By contrast, no significant correlation was observed between AGE, HE, and SP02 and adherence to MD. The presence or absence of a correlation between variables is consistent with the results obtained from the one-way ANOVA comparisons established between cohorts according to the level of adherence to MD (low, medium, or high). 

Regarding anthropometric variables, the correlations between these variables and greater adherence to the Mediterranean diet are consistent with studies carried out by Galan-Lopez et al. (2020) [36] with adolescents, by Baydemir et al. (2018) with university students, by Marcos-Pardo et al. (2020) [42] with middle-aged and older adults, and by Shatwan et al. (2021) [43] with adults in Gulf countries aged 20–55. Nevertheless, some studies conducted with schoolchildren and adolescents did not find an association between anthropometric parameters and adherence to MD [38,65]. Carillo López et al. (2018) [65] consider that the lack of correlation is due to factors such as the practice of physical activity, maternal obesity, and dietary beliefs that could exert a greater influence than adherence to MD. Similarly, Alacid et al. (2014) [65] and Vaquero-Cristóbal et al. (2018) [66] conducted studies with adolescent kayakers and paddlers and found no correlation between anthropometric parameters and adherence to MD [38]. Peláez Barrios et al. (2018) [38] corroborated these findings through a systematic review wherein most studies did not find correlations in children and adolescent athletes. Based on these results, we consider that the lack of correlation in the last three studies is caused by the use of small and homogeneous samples [38,67]. Additionally, it should also be noted that all the previously mentioned studies where no relationship was found between adherence to MD and anthropometric variables were carried out with subjects of growing age. Therefore, it cannot be ruled out that this factor may have conditioned the results. 

Furthermore, it should again be recalled that most of the studies conducted to date to analyze the adherence to MD have examined only a small number of anthropometric parameters, particularly BMI. Therefore, it is recommended that future studies include a comprehensive number of anthropometric variables. As for the present study, it was verified that while BM, BMI, %FAT, and AG have a strong correlation with adherence to MD, this correlation is weak in the case of WHR. This suggests that WHR—although it may be a better predictor for cardiovascular or metabolic disease than BMI, or AG [68]—could be a worse predictor than the rest of the anthropometric variables examined to identify whether young adults are eating healthily. Based on the results of the multiple regression analysis, BMI and %FAT would be the best predictor variables. However, further studies are required to confirm this finding. Finally, it is also important to mention that non-adherence to the Mediterranean diet is likely to cause those anthropometric parameters are not within the healthy range through two different mechanisms. The first one is excessive caloric intake, and the second is the alteration of the intestinal microbiota [69].

As expected, study participants with higher levels of MD adherence showed lower SBP and DBP values. These results agree somewhat with the findings of De Pergola and D’Alessandro (2018) [70]. After conducting a review study that included observational, intervention, meta-analyses, and systematic review studies, these authors concluded that, in general terms, the current scientific evidence indicates that MD has favorable effects on reducing BP. However, they also point out that there is insufficient data to determine the strength of this effect. Similarly, Magriplis et al. (2020) [71] found that adherence to MD reduces cardiovascular risk, especially in overweight and obese individuals. Interestingly, various studies have found that the reduction in BP is related to the intake of the main nutrients or food groups of the Mediterranean diet (i.e., olive oil, whole grains, plant-based proteins, and mono- and polyunsaturated fatty acids). Tigheet et al. (2010) [72] point out that the intake of whole grains reduces cardiovascular risk through blood pressure-lowering mechanisms, which in turn are related to weight management, increased potassium levels, reduced risk of insulin resistance, and reduced damage to blood vessels.

According to the patterns established by the Mediterranean diet, protein consumption implies increasing the intake of fish, nuts, and low-fat dairy and reducing the intake of red meat. However, this fact does not increase sodium intake, meaning that it positively affects BP. Jennings et al. (2019) [73], Teunissen-Beekman (2012) [74], and Tielemans et al. (2014) [75] also verified that high protein intake or protein supplementation is useful for decreasing blood pressure. Furthermore, Jennings et al. (2019) [73] state that omega-3 fatty acids help reduce inflammation and oxylipins (a compound that constricts blood vessels and increases blood pressure), which results in a reduction in BP. Similarly, Davis et al. (2017) [76], De Pergola and D’Alessandro (2018) [70], and Esposito et al. (2004) [77] consider that the endothelial function of blood vessels is favored by the consumption of MUFAs and olive oil due to their antioxidant content. Regarding the DP, the study participants of the present research with higher levels of adherence to MD obtained lower values. This result agrees with the study of Marcos-Pardo et al. (2020) [42] with middle-aged and older adults. However, the effect of adherence to MD on this variable has not been assessed in most of the studies conducted to date.

GLU also maintained a significant correlation with the levels of adherence to the Mediterranean diet in the present study. These results —which coincide with previous research [78]—could be related to the consumption of olive oil and monounsaturated fatty acids since both have been associated with lower GLU [79], improved insulin sensitivity, and lower resistance to insulin [80,81]. These data reinforce the importance of following a Mediterranean diet to avoid non-communicable diseases with high mortality rates such as diabetes and cardiovascular diseases because both are related to high GLU [82,83,84].

Interestingly, the present study’s results also showed significant correlations between anthropometric (BM, BMI, %FAT, LEAN, AG, and WHR) and health variables (SBP, DBP, DP, and GLU). These interconnections reveal the possibility that the human organism works as a functional unit so that the different systems that make up the human being interact with each other, and the correct functioning of one organ conditions the health of the others. This circumstance highlights the usefulness of the mentioned variables for evaluating the health status, health risks, and the diagnosis and monitoring of diseases [85].

Furthermore, three variables did not correlate with adherence to MD (HE, AGE, and SPO2). As for the AGE, the results of the present study are compatible with most of the previous research conducted with adults, wherein AGE did not significantly determine the levels of adherence to MD [27]. Moreover, previous studies have verified that malnutrition can impact HE and even cause rickets [86,87]. However, we understand that this only occurs when there is a severe nutritional deficit involving a significant deficiency of vitamin D, calcium, and phosphorus [87]. SPO2 also seems to be unaffected by the levels of adherence to MD. This result agrees with the current scientific evidence, which indicates that diet is not one of the main factors conditioning the SPO2 levels [88]. 

Finally, it is necessary to mention the strengths and limitations of the present research. As for the strengths, the study was conducted with a large stratified sample of subjects, in a non-Mediterranean country, and with an age group that—despite not being extensively studied—is interesting to analyze since college-aged individuals are often subject to modifications in their habits and schedules, which may affect their eating patterns. Likewise, a broad set of anthropometric and health parameters was analyzed. Regarding the limitations, women were not included in the study, and the lipid profile was not analyzed. Therefore, it is recommended that future studies include both sexes and different population groups and analyze as many biomarkers as possible, including the lipid profile. Additionally, the KIDMED questionnaire was administered using the digital support of Google Forms to avoid incomplete or partial submissions. However, this circumstance can also be considered a limitation of the study because that is not the format initially envisaged for the questionnaire management.

## 5. Conclusions

Greater adherence to Mediterranean diet is associated with healthier values of numerous anthropometric and health variables (body mass, body mass index, body fat percentage, lean mass, systolic blood pressure, diastolic blood pressure, fasting blood glucose, abdominal girth, waist-to-hip ratio, and double product). In contrast, no significant correlation was observed between age, height, and Oxygen saturation and adherence to Mediterranean diet. Many anthropometric parameters (body mass, body mass index, body fat percentage, lean mass, abdominal girth, and waist-to-hip ratio) present significant correlations with health variables (systolic blood pressure, diastolic blood pressure, double product, and fasting blood glucose). This could be useful for medical diagnosis, health monitoring, and health risks detection. Based on the levels of adherence to Mediterranean diet and the KIDMED index found in the present research, there is significant room for improvement in the dietary habits of college-aged citizens residing in Riyadh. Therefore, considering these results and the prevalence of obesity in the Middle East, nutritional interventions should be urgently implemented with the target population of this study to prevent nutrition-related diseases and promote public health.

## Figures and Tables

**Table 1:** Characteristics of the participants and results obtained in the anthropometric and health assessments.

Variable	$\bar{X}$	SD
AGE (years)	19.10	2.59
WE (kg)	79.22	22.36
HE (cm)	176.22	6.07
BMI (Kg/m^2^)	26.40	6.76
FAT (%)	22.01	7.28
LBM (kg)	60.42	8.99
AG (cm)	86.81	13.17
WHR (index)	0.85	0.06
SBP (mmHg)	130.02	13.28
DBP (mmHg)	75.06	9.06
SPO2 (%)	97.31	4.72
DP (mmHg × HR)	8845.44	1245.57
GLU (mg/dL)	99.95	6.12

Legend: WE: weight; HE: height; BMI: body mass index; FAT: body fat percentage; LBM: lean body mass; AG: abdominal Girth; WHR: waist-to-hip ration; SBP: systolic blood pressure, DBP: diastolic blood pressure; SPO2: Oxygen saturation; DP: double product; GLU: fasting blood glucose.

**Table 2:** Correlation matrix: correlation between variables.

		KIDMED	AGE	BM	HE	BMI	%FAT	LEAN	SPO2	SBP	DBP	GLU	AG	WHR
AGE	*r*	−0.029												
	*p*	0.524												
BM	*r*	−0.571 *	0.089 *							Strength of the association				
	*p*	<0.001	0.047									Null		
HE	*r*	0.053	0.005	−0.052								Very weak		
	*p*	0.243	0.905	0.245								Weak		
BMI	*r*	−0.614 *	0.067	0.925 *	−0.051							Moderate		
	*p*	<0.001	0.138	<0.001	0.26							Strong		
%FAT	*r*	−0.558 *	−0.019	0.741 *	−0.028	0.815 *						Very strong		
	*p*	<0.001	0.668	<0.001	0.534	<0.001								
LEAN	*r*	−0.497 *	0.151*	0.943 *	−0.063	0.797 *	0.558 *							
	*p*	<0.001	0.001	<0.001	0.159	<0.001	<0.001							
SPO2	*r*	0.083	−0.015	−0.023	0.018	−0.058	−0.015	−0.014						
	*p*	0.066	0.732	0.606	0.69	0.197	0.738	0.748						
SBP	*r*	−0.238 *	0.086	0.274 *	−0.05	0.288 *	0.230 *	0.269 *	−0.037					
	*p*	<0.001	0.056	<0.001	0.268	<0.001	<0.001	<0.001	0.415					
DBP	*r*	−0.217 *	0.209 *	0.320 *	−0.06	0.325 *	0.220 *	0.322 *	−0.046	0.291 *				
	*p*	<0.001	<0.001	<0.001	0.186	<0.001	<0.001	<0.001	0.307	<0.001				
GLU	*r*	−0.407 *	−0.015	0.558 *	−0.032	0.619 *	0.605 *	0.450 *	−0.063	0.214 *	0.134 *			
	*p*	<.001	0.738	<0.001	0.481	<0.001	<0.001	<0.001	0.161	<0.001	0.003			
AG	*r*	−0.564 *	0.079	0.887 *	−0.058	0.912 *	0.760 *	0.777 *	−0.058	0.279 *	0.305 *	0.568 *		
	*p*	<0.001	0.079	<0.001	0.199	<0.001	<0.001	<0.001	0.194	<0.001	<0.001	<0.001		
WHR	*r*	−0.090 *	0.132 *	0.144 *	−0.002	0.224 *	0.193 *	0.07	−0.073	0.142 *	0.087	0.158 *	0.414 *	
	*p*	0.045	0.003	0.001	0.956	<0.001	<0.001	0.119	0.106	0.002	0.054	<0.001	<0.001	
DP	*r*	−0.265 *	−0.060	0.446 *	0.043	0.480 *	0.410 *	0.405 *	−0.042	0.799 *	0.283 *	0.307 *	0.453 *	0.143 *
	*p*	<0.001	0.186	<0.001	0.345	<0.001	<0.001	<0.001	0.355	<0.001	<0.001	<0.001	<0.001	0.001

Legend: r: Pearson correlation; p: level of significance; *: significant correlation found; KIDMED: Mediterranean diet index; BM: body mass; BMI: body mass index; %FAT: body fat percentage; LEAN: lean mass; SPO2: Oxygen saturation; SBP: systolic blood pressure; DBP: diastolic blood pressure; GLU: fasting blood glucose; AG: abdominal girth; WHR: waist-to-hip ratio. DP: double product.

**Table 3:** Stepwise multiple linear regression analysis of the relation between KIDMED test anthropometric and health variables.

Analysis	Dependent Variable	Included Independent Variable/s	R^2^	*p*-Value	Standardized Coefficient (β)	*p*-Value
Model 1	KIDMED	BMI	0.23	<0.001	−0.48	<0.001
Model 2	KIDMED	BMI FAT	0.24	0.014	−0.35 −0.17	<0.001 <0.001

Legend: BMI: body mass index; %FAT: body fat percentage.

## Data Availability

Not applicable.
